# A Sure Cure for Pneumonia

**Published:** 1917-02

**Authors:** 


### A Sure Cure for Pneumonia.

Marcus A. Redding, of Los Angeles, declares the following prescription has never failed to relieve and cure patients suffering from pneumonia.

The remedy is: Saturate a ball of cotton one inch in diameter with spirits of grain alcohol, add three drops of chloroform to each ball of cotton; place it between the patient's teeth (after first using vaseline on the gums to prevent burning) and let the patient inhale the fumes in long, deep breaths for fifteen minutes, then rest for fifteen minutes or more; inhale again and repeat the above for twenty times.

The result will be that the lungs will relax and expand to their normal condition; in twenty-four hours the patient is out of danger and in forty-eight hours cured, although weak.

Change the cotton every seven minutes, else the saliva will dilute the alcohol.

---

The Phenix Sanitarium of Colorado, Texas, has been sold to Drs. T. J. and J. D. Ratliff.

